# Risk Factors for Long-Term Vancomycin-Resistant Enterococci Persistence—A Prospective Longitudinal Study

**DOI:** 10.3390/microorganisms7100400

**Published:** 2019-09-26

**Authors:** Carlos L. Correa-Martinez, Verena B. Stollenwerk, Annelene Kossow, Frieder Schaumburg, Alexander Mellmann, Stefanie Kampmeier

**Affiliations:** 1 Institute of Hygiene, University Hospital Münster, Robert-Koch-Straße 41, 48149 Münster, Germany; Carlos.Correa@ukmuenster.de (C.L.C.-M.); Verena.Stollenwerk@ukmuenster.de (V.B.S.); Annelene.Kossow@ukmuenster.de (A.K.); Alexander.Mellmann@ukmuenster.de (A.M.); 2 Institute of Medical Microbiology, University Hospital Münster, Domagkstraße 10, 48149 Münster, Germany; Frieder.Schaumburg@ukmuenster.de

**Keywords:** vancomycin-resistant enterococci (VRE), persistence, risk factors, whole genome sequencing

## Abstract

Vancomycin-resistant enterococci (VRE) are important nosocomial pathogens that require effective infection control measures, representing a challenge for healthcare systems. This study aimed at identifying risk factors associated with prolonged VRE carriage and determining the rate of clearance that allows the discontinuation of contact precautions. During a 2-year study, screening was performed in patients with a history of VRE or at risk of becoming colonized. After bacterial identification and antibiotic susceptibility testing, glycopeptide resistance was confirmed by PCR. Isolates were compared via whole genome sequence-based typing. Risk factors were recorded, and follow-up screening was performed upon readmission, defining patients as long-term carriers if still colonized ≥10 weeks after first detection. Of 1059 patients positive for VRE, carriage status was assessed upon readmission in 463 patients. VRE was cleared in 56.4% of the cases. Risk factors associated with long-term persistence were hospital stays (frequency, length), hemato-oncological disease, systemic treatment with steroids, and use of antibiotics. No specific genotypic clustering was observed in patients with VRE clearance or persistence. VRE clearance is possibly underestimated. The identification of risk factors favoring long-term carriage may contribute to a targeted implementation of infection control measures upon readmission of patients with history of VRE.

## 1. Introduction

Three decades after their emergence in Europe [1,2], vancomycin-resistant enterococci (VRE) were classified by the World Health Organization in 2017 as microorganisms with a high level of priority regarding the research and development of new and effective antibiotics [3]. The increasing incidence of invasive infections caused by VRE represents a public health challenge in Germany [4] as well as in several regions worldwide [5]. Additionally, cross-genus horizontal gene transfer observed between VRE and methicillin-resistant *Staphylococcus aureus* (MRSA) represents a major concern as the transmission of *vanA* and *cfr* genes has been shown to render resistance against vancomycin and linezolid to MRSA strains [6,7,8]. The risk for the spread of VRE is highest in the healthcare setting [9], partially due to a high proportion of patients either being already colonized [10,11] or displaying risk factors that favor this condition, e.g., comorbidities, immunosuppressive diseases, or steroid or antibiotic treatment [12,13,14,15,16,17]. Several studies have demonstrated the relationship between the hospital-wide use of specific antimicrobials and the increasing incidence of nosocomial VRE transmission [15,18,19]. The constant flux of VRE patients and healthcare workers through different areas of a healthcare facility [20,21], as well as numerous surfaces and objects capable of acting as fomites also facilitate transmission in this setting [11,22,23]. Strategies of infection control and prevention have been developed globally [24], aiming at reducing the transmission of VRE. Although the gut is considered to be the main reservoir in humans, decolonization regimens are not feasible yet. Instead, bundles of measures that passively minimize the risk of transmission are adopted. Within the German healthcare system, these strategies focus on the detection of VRE carriers by screening for rectal colonization, the establishment of contact precautions, the disinfection of potentially contaminated surfaces and elements present in the environment, as well as the implementation of antibiotic stewardships programs as a preventive approach [25,26]. Although such measures have proven to be effective in containing the spread of multiresistant microorganisms, they are cost and time intensive, requiring trained personnel and a high compliance for successful implementation [27]. Contact precautions may be discontinued once the clearance of colonization with VRE has been documented, indicated by negative results on at least three consecutive occasions, greater than or equal to one week apart. This definition was established by the Centers for Disease Control and Prevention in 1995 [28], and has since then been broadly adopted in infection control standards, including the national German guidelines on prevention of infections with multidrug-resistant enterococci [26]. Since VRE colonization is generally thought to persist chronically in most patients, contact precautions are usually maintained over long periods [29]. The detection of VRE clearance allows for the discontinuation of all infection control measures, therefore requiring a close assessment of the VRE status by performing periodical follow-up screenings. We hypothesize that the number of patients with spontaneous VRE clearance is currently underestimated as recommendations for regular assessment of colonization are generally lacking. We therefore elucidated, here, the real proportion of long-term VRE carriage and analyzed risk factors associated with long-term VRE carriage.

## 2. Materials and Methods

### 2.1. Clinical Setting and Infection Control Measures

The 1527-bed University Hospital Münster (UHM) is a tertiary care center, admitting ca. 65,000 patients every year. In the daily routine, screening for multidrug-resistant organisms is performed for methicillin-resistant *Staphylococcus aureus* (MRSA) and multidrug-resistant Gram-negative bacteria according to the national German guidelines [30,31]. A standardized VRE screening is carried out in patients at high risk of developing VRE infections (i.e., hemato-oncological patients) or in patients with a history of VRE colonization and/or infection. This screening policy, established in the beginning of 2016, is in accordance with the national German guidelines published in October 2018 [26]. In case of VRE detection (colonization/infection), extended hygiene measures include contact isolation of the VRE patient in a separate room with separate sanitary facilities. Staff members and visitors are advised to wear personal protective equipment consisting of gloves and gowns. Surface disinfection is performed at least once a day. Patient isolation can be stopped if three anorectal swabs, taken at least one week apart, are negative for VRE providing that patients are not under antibiotic treatment during this time.

### 2.2. Detection of VRE Long-Term Carriers

During a two-year period (October 2016–October 2018) VRE positive patients admitted to the UHM were recruited. Patients who were not readmitted after the initial VRE diagnosis were excluded, since the evolution of the VRE status could not be assessed in these cases. Swabs taken from these patients during the first hospital stay or after readmission were collected. In the absence of established definitions, patients were classified as “VRE long-term carriers” if rectal colonization with the same VRE genotype was still present ≥10 weeks after first VRE detection in rectal swabs [32]. In parallel, risk factors known to favor VRE colonization [11,12,13,14,15,16,17] were recorded. These included age, gender, overall length of stay, number of admissions, average length of stay per admission, number of stays, comorbidities, immunosuppressive diseases, steroid treatment, admission from another hospital or ICU, and antibiotic treatment, especially concentrating on frequently prescribed drugs known to select enterococci or VRE [12,13,15,33,34].

### 2.3. VRE Swab Samples, Culturing, Antibiotic Susceptibility Testing, PCR Testing

Swabs were obtained rectally (5 cm ab ano) (Transwab^®^ m40 compliant, mwe, Corsham, Wiltshire, UK) and subsequently streaked onto chromogenic selective agar (VRESelect^TM^, Biorad, München, Germany). Species identification of suspected colonies (pink or blue) was performed with MALDI-TOF MS (Bruker Corporation, Bremen, Germany). In accordance with the current European Committee on Antimicrobial Susceptibility Testing (EUCAST) standards for clinical breakpoints [35], susceptibility testing was performed using the VITEK^®^ 2 system (bioMérieux, Nürtingen, Germany). Confirmation of species identification and glycopeptide resistance (*vanA*, *vanB*, *vanC1*, and *vanC2/3*) was done using the GenoType *Enterococcus*^®^ line probe (Hain Lifescience, Nehren, Germany). In addition, the presence of *van* genes in the tested isolates was confirmed by the whole genome sequencing (WGS) data.

### 2.4. Whole Genome Sequence-Based Typing

To uncover genetic relationships of the VRE strains, isolates were compared via WGS-based typing using the Illumina MiSeq platform (Illumina Inc., San Diego, CA, USA) [36]. After quality trimming, coding core genome regions were compared in a gene-by-gene approach (core genome multilocus sequence typing, cgMLST) using the SeqSphere+ software version 6.0.0 (Ridom GmbH, Münster, Germany) and the published *E. faecium* cgMLST target scheme [37]. To display the clonal relationship of genotypes, the minimum spanning tree algorithm was applied using the same software. Genotypes differing in ≤3 alleles were rated as closely related. For backwards compatibility with classical molecular typing (i.e., multilocus sequence typing (MLST)), the MLST sequence types (STs) were extracted from the WGS data in silico.

### 2.5. Statistical Analysis

All data were expressed as absolute numbers or percentages. Descriptive statistics were performed using the IBM SPSS Statistics software (Armonk, NY, USA). For risk factor analysis, chi-square or Fisher’s exact test were used for categorical data as appropriate. Normally distributed continuous variables were compared using the two-sided Student’s *t*-test. Statistical significance was set at *p* < 0.05.

## 3. Results

### 3.1. VRE Persistence and Associated Risk Factors

In total, 1059 VRE patients were admitted to the UHM. Of these, 134 had a pre-existing VRE medical history, while in the remaining 925 patients, VRE was newly detected. Follow-up results at least 10 weeks after initial detection of VRE colonization/infection were available for 463 patients. Of these, 202 (43.6%) patients were still VRE-positive, thus defining the status of VRE persistence, while VRE clearance was determined in the remaining 261 patients (56.4%). Clinical characteristics favoring VRE persistence are summarized in Table 1. Long-term carriers were significantly older and suffered from hemato-oncological diseases. Moreover, they had more frequent and longer hospital stays and received antibiotic agents more often and for a longer time. The antibiotic substances piperacillin/tazobactam, ceftriaxone, clindamycin, trimethoprim/sulfamethoxazole, ciprofloxacin, and vancomycin were significantly associated with VRE persistence.

### 3.2. VRE Genotypes and Genetic Distribution of Strains

**Figure 1:**
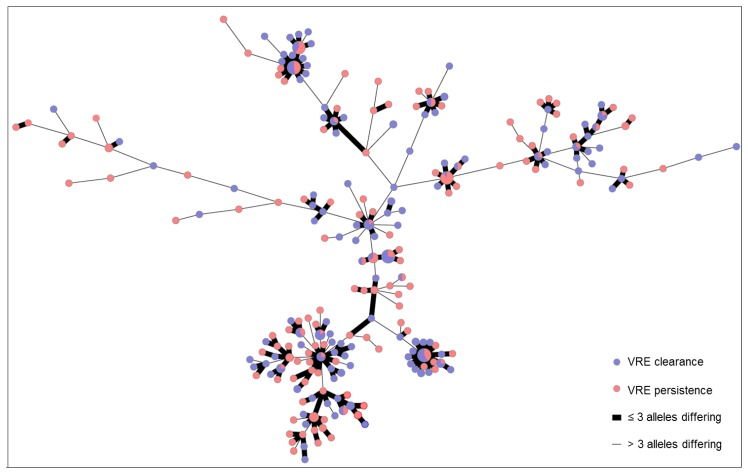
Minimum spanning tree of VRE isolates illustrating their genotypic relationship, Münster University Hospital, 2016–2018. VRE strains (365) isolated from patients presenting VRE clearance (*blue*) and VRE persistence (*red*), based on 1423 core genome multilocus sequence typing (cgMLST) target genes, pairwise, ignoring missing values. Size of dots correlates with the number of identical genotypes. Thickness of the connecting line indicates the genetic similarity between different genotypes.

Table 2 shows the *van* genotypes in patients with VRE clearance and persistence in detail. Out of 463 patients, ca. 71% of all detected strains harbored *vanB*. Of all isolates derived from long-term and non-long-term carriers, 365 isolates were available for WGS. The following STs were detected among these samples: ST117 (60.8%; 222 isolates), ST262 (12.6%, 46), ST203 (11.5%, 42), ST80 (6.0%, 22), ST721 (4.9%; 18), ST78 (1.9%, 7), ST192 (0.8%; 3), and ST17 (0.5%; 2). Three isolates (0.8%) could not be correctly typed. cgMLST-based typing resulted in several clusters of genetically closely related genotypes (Figure 1). There was no specific clustering of isolates associated with VRE persistence or VRE clearance.

## 4. Discussion

VRE are a rising problem worldwide. While previous studies have analyzed the risk factors for VRE acquisition, we observed the temporal evolvement of VRE eventually leading to persistence or clearance.

By prospectively following patients with VRE detection at admission, we surprisingly found that 56.4% of patients spontaneously lost VRE 10 weeks after first detection. In contrast to other studies, which describe a rate of persistence of approximately 80% [38,39], only 43.6% of our patients were identified as long-term carriers. *vanB*-positive strains were more common among both persistent carriers (70.3%) and patients with clearance (71.3%), concurring with the national trend in Germany [40]. cgMLST analysis of the VRE strains revealed no specific genetic pattern that favors VRE persistence or clearance. Hence, host-associated factors and treatment strategies are very likely the main factors determining the VRE carrier status.

We found that factors strongly associated with VRE persistence are similar to those known to favor colonization, such as hemato-oncological diseases, long and multiple hospital stays, and steroid and antibiotic therapy [3,4,12,14,17,32,41]. Dialysis, a further factor described to favor VRE colonization [32,33], was not shown to be a risk factor for VRE carriage persistence in our study. Moreover, regiments using broad spectrum antibiotics like third generation cephalosporines increased the likelihood of VRE persistence, possibly due to a disruption of the normal gut flora, allowing the selective proliferation of VRE, as previously stated [42]. Fortunately, this risk factor can be modified: the implementation of institutional policies for rational use of antibiotics, such as antibiotic stewardship programs, represent a valuable preventive strategy to tackle the VRE problem as reported in studies showing a significant reduction of colonization and infection with antibiotic-resistant bacteria as a direct result of such approaches [34]. Moreover, the constant emergence of new bacterial resistance mechanisms calls for the development of new antimicrobial substances. Recent studies have described novel compounds that could be potentially employed in the treatment of infections with vancomycin-resistant enterococci [43,44].

In contrast to MRSA, there are no eradication approaches for VRE. Therefore, infection control bundle strategies rely solely on screening and isolation of colonized patients. Since screening is cost-intensive [3] and isolation of patients is associated with reduced quality of care [45], a risk stratification upon admission of patients with history of VRE would allow to identify those likely to be cleared of colonization, thus not requiring them to be isolated. This would lead to a reduction of unnecessary contact precautions and costs for microbiological diagnostic procedures.

Our study has some limitations. First, our results do not reveal causality but correlations between certain risk factors and the persistence of VRE carriage. Second, we did not consider the patients’ environment, which have also been suggested to influence the risk of VRE colonization [24,46]. However, neither of these limitations hindered the achievement of the study’s main objectives, namely, the determination of the VRE clearance rate and the identification of risk factors significantly associated with VRE persistence.

## 5. Conclusions

Infection control management of VRE positive patients is linked to increased use of human and financial resources. Only approximately every second VRE patient becomes a long-term carrier. Hence, the identification of patient-associated risk factors may be helpful in predicting the VRE carriage, allowing for infection control measures as screening and contact precautions to focus on patients at increased risk for VRE persistence.

## Figures and Tables

**Table 1:** Comparison of clinical characteristics of patients with vancomycin-resistant enterococci (VRE)-persistence and VRE-clearance, Münster University Hospital, 2016–2018.

Clinical Characteristic	VRE-Persistence (*n* = 202)	VRE-Clearance (*n* = 261)	*p*-Value
Sex (male)	57.4%	60.5%	0.50
Age (years)	59.7 (range 1.8–93.1)	55.7 (range 0.6–97.7)	0.02
Overall length of stay (days)	76.4 (range: 1–478)	37.8 (range 1–216)	0.001
Average length of stay per admission (days)	35.3 (1–340)	20.1 (1–188)	0.001
Number of stays	3.3 (range: 1–29)	2.3 (range: 1–10)	0.001
Hemato-oncological disease	119 (58.9%)	122 (46.7%)	0.01
Liver insufficiency	41 (20.3%)	43 (16.5%)	0.29
Liver transplantation	9 (4.5%)	22 (8.4%)	0.09
Kidney insufficiency	88 (43.6%)	114 (43.7%)	0.98
Dialysis	37 (18.3%)	35 (13.4%)	0.15
Immunosuppressive disease	143 (70.8%)	169 (64.8%)	0.17
Inflammatory bowel disease	2 (1.0%)	5 (1.9%)	0.47
Rheumatic disease	1 (0.5%)	5 (1.9%)	0.24
Granulomatosis with polyangiitis	1 (0.5%)	1 (0.4%)	1.00
HIV	1 (0.5%)	2 (0.8%)	0.58
Systemic lupus erythematosus	1 (0.5%)	1 (0.4%)	1.00
Idiopathic thrombocytic purpura	0 (0.0%)	1 (0.4%)	1.00
Transplantation	20 (10.0%)	39 (14.9%)	0.13
Multiple sclerosis	1 (0.5%)	1 (0.4%)	1.00
Systemic scleroderma	1 (0.5%)	0 (0.0%)	0.44
Antibiotics	191 (94.6%)	222 (85.1%)	0.001
Penicillin	9 (4.5%)	8 (3.1%)	0.46
Ampicillin	12 (5.9%)	8 (3.1%)	0.17
Amoxicillin	5 (2.5%)	6 (2.3%)	1.00
Ampicillin/sulbactam	14 (6.9%)	20 (7.7%)	0.86
Amoxicillin/clavulanic acid	17 (8.4%)	16 (6.1%)	0.37
Flucloxacillin	18 (8.9%)	18 (6.9%)	0.48
Piperacillin/tazobactam	145 (71.3%)	134 (51.3%)	<0.001
Cefuroxime	19 (9.4%)	24 (9.2%)	1.00
Ceftriaxone	43 (21.3%)	36 (13.8%)	0.03
Ciprofloxacin	109 (54.0%)	76 (29.1%)	<0.001
Erythromycin	9 (4.5%)	10 (3.8%)	0.82
Clindamycin	35 (17.3%)	21 (0.8%)	0.004
Colistin	8 (4.0%)	3 (0.1%)	0.06
Trimethoprim/sulfamethoxazole	102 (50.5%)	88 (33.7%)	<0.001
Metronidazole	20 (9.9%)	18 (6.9%)	0.31
Rifampicin	17 (8.4%)	18 (6.9%)	0.60
Fosfomycin	10 (5.0%)	10 (3.8%)	0.65
Vancomycin	92 (45.5%)	59 (22.6%)	<0.001
Duration of treatment with antibiotics (days)	97.7 (range: 0–430)	25.0 (range: 0–372)	<0.001
Systemic steroids	121 (59.9%)	116 (44.4%)	0.001
Admission from another hospital	62 (30.7%)	60 (23%)	0.06
Admission from other ICUs	2 (1.0%)	9 (3.4%)	0.09

**Table 2:** Number of Vancomycin-resistant enterococci (VRE) patients and genotype distribution stratified by VRE persistence and VRE clearance, Münster University Hospital, 2016–2018.

Colonization Status	No. of Patients with *Van* Genotype (%)				
	*vanA*	*vanB*	*vanA* + *vanB*	No Genotype Data Available	Total
**VRE-persistence**	58 (28.7%)	142 (70.3%)	2 (1.0%)	0 (0.0%)	202 (43.6%)
**VRE-clearance**	64 (24.5%)	186 (71.3%)	2 (1.0%)	9 (3.4%)	261 (56.4%)
**Total**	122 (26.3%)	328 (70.8%)	4 (1.0%)	9 (1.9%)	463 (100%)

## References

[1] Uttley A.H., Collins C.H., Naidoo J., George R.C. (1988). Vancomycin-resistant enterococci. Lancet.

[2] Leclercq R., Derlot E., Duval J., Courvalin P. (1988). Plasmid-mediated resistance to vancomycin and teicoplanin in *Enterococcus faecium*. N. Engl. J. Med..

[3] Tacconelli E., Carrara E., Savoldi A., Harbarth S., Mendelson M., Monnet D.L., Pulcini C., Kahlmeter G., Kluytmans J., Carmeli Y. (2018). Discovery, research, and development of new antibiotics: The WHO priority list of antibiotic-resistant bacteria and tuberculosis. Lancet Infect. Dis..

[4] Remschmidt C., Schröder C., Behnke M., Gastmeier P., Geffers C., Kramer T.S. (2018). Continuous increase of vancomycin resistance in enterococci causing nosocomial infections in Germany−10 years of surveillance. Antimicrob. Resist. Infect. Control.

[5] Pfaller M.A., Cormican M., Flamm R.K., Mendes R.E., Jones R.N. (2019). Temporal and Geographic Variation in Antimicrobial Susceptibility and Resistance Patterns of Enterococci: Results from the SENTRY Antimicrobial Surveillance Program, 1997–2016. Open Forum Infect. Dis..

[6] Cafini F., Nguyen le T.T., Higashide M., Roman F., Prieto J., Morikawa K. (2016). Horizontal gene transmission of the *cfr* gene to MRSA and *Enterococcus*: Role of *Staphylococcus epidermidis* as a reservoir and alternative pathway for the spread of linezolid resistance. J. Antimicrob. Chemother..

[7] Juhas M. (2015). Horizontal gene transfer in human pathogens. Crit. Rev. Microbiol..

[8] Willems R.J., Top J., Marga van Santen D., Coque T.M., Baquero F., Grundmann H., Bonten M.J. (2005). Global spread of vancomycin-resistant *Enterococcus faecium* from distinct nosocomial genetic complex. Emerg. Infect. Dis..

[9] Zhou M.J., Li J., Salmasian H., Zachariah P., Yang Y.X., Freedberg D.E. (2019). The local hospital milieu and healthcare-associated vancomycin-resistant enterococcus acquisition. J. Hosp. Infect..

[10] Bonten M.J., Slaughter S., Ambergen A.W., Hayden M.K., van Voorhis J., Nathan C., Weinstein R.A. (1998). The role of “colonization pressure” in the spread of vancomycin-resistant enterococci: An important infection control variable. Arch. Intern. Med..

[11] Ford C.D., Lopansri B.K., Gazdik M.A., Webb B., Snow G.L., Hoda D., Adams B., Petersen F.B. (2016). Room contamination, patient colonization pressure, and the risk of vancomycin-resistant *Enterococcus* colonization on a unit dedicated to the treatment of hematologic malignancies and hematopoietic stem cell transplantation. Am. J. Infect. Control.

[12] Zacharioudakis I.M., Zervou F.N., Ziakas P.D., Rice L.B., Mylonakis E. (2015). Vancomycin-resistant enterococci colonization among dialysis patients: A meta-analysis of prevalence, risk factors, and significance. Am. J. Kidney Dis..

[13] Papadimitriou-Olivgeris M., Drougka E., Fligou F., Kolonitsiou F., Liakopoulos A., Dodou V., Anastassiou E.D., Petinaki E., Marangos M., Filos K.S. (2014). Risk factors for enterococcal infection and colonization by vancomycin-resistant enterococci in critically ill patients. Infection.

[14] D’Agata E.M., Green W.K., Schulman G., Li H., Tang Y.W., Schaffner W. (2001). Vancomycin-resistant enterococci among chronic hemodialysis patients: A prospective study of acquisition. Clin. Infect. Dis..

[15] Remschmidt C., Behnke M., Kola A., Pena Diaz L.A., Rohde A.M., Gastmeier P., Schwab F. (2017). The effect of antibiotic use on prevalence of nosocomial vancomycin-resistant enterococci—An ecologic study. Antimicrob. Resist. Infect. Control.

[16] Pan S.C., Wang J.T., Chen Y.C., Chang Y.Y., Chen M.L., Chang S.C. (2012). Incidence of and risk factors for infection or colonization of vancomycin-resistant enterococci in patients in the intensive care unit. PLoS ONE.

[17] Ford C.D., Lopansri B.K., Haydoura S., Snow G., Dascomb K.K., Asch J., Bo Petersen F., Burke J.P. (2015). Frequency, risk factors, and outcomes of vancomycin-resistant *Enterococcus* colonization and infection in patients with newly diagnosed acute leukemia: Different patterns in patients with acute myelogenous and acute lymphoblastic leukemia. Infect. Control Hosp. Epidemiol..

[18] Carmeli Y., Eliopoulos G.M., Samore M.H. (2002). Antecedent treatment with different antibiotic agents as a risk factor for vancomycin-resistant *Enterococcus*. Emerg. Infect. Dis..

[19] Patterson J.E. (2001). Antibiotic utilization: Is there an effect on antimicrobial resistance?. Chest.

[20] Ott M., Wirick H. (2008). Vancomycin-resistant enterococci (VRE) and the role of the healthcare worker. Can. Oper. Room Nurs. J..

[21] Jackson S.S., Harris A.D., Magder L.S., Stafford K.A., Johnson J.K., Miller L.G., Calfee D.P., Thom K.A. (2019). Bacterial burden is associated with increased transmission to health care workers from patients colonized with vancomycin-resistant *Enterococcus*. Am. J. Infect. Control.

[22] Suleyman G., Alangaden G., Bardossy A.C. (2018). The Role of Environmental Contamination in the Transmission of Nosocomial Pathogens and Healthcare-Associated Infections. Curr. Infect. Dis. Rep..

[23] Chemaly R.F., Simmons S., Dale C., Ghantoji S.S., Rodriguez M., Gubb J., Stachowiak J., Stibich M. (2014). The role of the healthcare environment in the spread of multidrug-resistant organisms: Update on current best practices for containment. Ther. Adv. Infect. Dis..

[24] De Angelis G., Cataldo M.A., De Waure C., Venturiello S., La Torre G., Cauda R., Carmeli Y., Tacconelli E. (2014). Infection control and prevention measures to reduce the spread of vancomycin-resistant enterococci in hospitalized patients: A systematic review and meta-analysis. J. Antimicrob. Chemother..

[25] Biehl L.M., Bertz H., Bogner J., Dobermann U.H., Kessel J., Kramer C., Lemmen S., von Lilienfeld-Toal M., Peter S., Pletz M.W. (2017). Screening and contact precautions—A survey on infection control measures for multidrug-resistant bacteria in German university hospitals. Antimicrob. Resist. Infect. Control.

[26] RKI (2018). Hygienemaßnahmen zur Prävention der Infektion durch Enterokokken mit speziellen Antibiotikaresistenzen. Empfehlung der Kommission für Krankenhaushygiene und Infektionsprävention (KRINKO) beim Robert-Koch-Institut. Bundesgesundheitsblatt.

[27] Sprague E., Reynolds S., Brindley P. (2016). Patient Isolation Precautions: Are They Worth It?. Can. Respir. J..

[28] Favero M.S., Gaynes R.P., Jarvis W.R., Shaw J., Tablan O.C., Tenover F.C. (1995). Recommendations for preventing the spread of vancomycin resistance; recommendations of the Hospital Infection Control Practices Advisory Committee (HICPAC). Am. J. Infect. Control.

[29] Shenoy E.S., Paras M.L., Noubary F., Walensky R.P., Hooper D.C. (2014). Natural history of colonization with methicillin-resistant *Staphylococcus aureus* (MRSA) and vancomycin-resistant *Enterococcus* (VRE): A systematic review. BMC Infect. Dis..

[30] Ruscher C. (2014). Empfehlungen zur Prävention und Kontrolle von Methicillin-resistenten Staphylococcus aureus-Stämmen (MRSA) in medizinischen und pflegerischen Einrichtungen. Bundesgesundheitsblatt Gesundh. Gesundh..

[31] Wendt C., Baum H., Kaase M., Meyer E., Suger-Wiedeck H., Ruscher C. (2012). Hygienemaßnahmen bei Infektionen oder Besiedlung mit multiresistenten gramnegativen Stäbchen: Empfehlung der Kommission für Krankenhaushygiene und Infektionsprävention (KRINKO) beim Robert Koch-Institut (RKI). Bundesgesundsheitsblatt.

[32] Sohn K.M., Peck K.R., Joo E.J., Ha Y.E., Kang C.I., Chung D.R., Lee N.Y., Song J.H. (2013). Duration of colonization and risk factors for prolonged carriage of vancomycin-resistant enterococci after discharge from the hospital. Int. J. Infect. Dis..

[33] Kampmeier S., Kossow A., Clausen L.M., Knaack D., Ertmer C., Gottschalk A., Freise H., Mellmann A. (2018). Hospital acquired vancomycin resistant enterococci in surgical intensive care patients—a prospective longitudinal study. Antimicrob. Resist. Infect. Control.

[34] Baur D., Gladstone B.P., Burkert F., Carrara E., Foschi F., Dobele S., Tacconelli E. (2017). Effect of antibiotic stewardship on the incidence of infection and colonisation with antibiotic-resistant bacteria and *Clostridium difficile* infection: A systematic review and meta-analysis. Lancet Infect. Dis..

[35] The European Committee on Antimicrobial Susceptibility Testing Breakpoint Tables for Interpretation of MICs and Zone Diameters. http://www.eucast.org/fileadmin/src/media/PDFs/EUCAST_files/Breakpoint_tables/v_9.0_Breakpoint_Tables.pdf.

[36] Mellmann A., Bletz S., Boking T., Kipp F., Becker K., Schultes A., Prior K., Harmsen D. (2016). Real-Time Genome Sequencing of Resistant Bacteria Provides Precision Infection Control in an Institutional Setting. J. Clin. Microbiol..

[37] De Been M., Pinholt M., Top J., Bletz S., Mellmann A., van Schaik W., Brouwer E., Rogers M., Kraat Y., Bonten M. (2015). Core Genome Multilocus Sequence Typing Scheme for High- Resolution Typing of *Enterococcus faecium*. J. Clin. Microbiol..

[38] Baden L.R., Thiemke W., Skolnik A., Chambers R., Strymish J., Gold H.S., Moellering R.C., Eliopoulos G.M. (2001). Prolonged colonization with vancomycin-resistant *Enterococcus faecium* in long-term care patients and the significance of “clearance”. Clin. Infect. Dis..

[39] Patel R., Allen S.L., Manahan J.M., Wright A.J., Krom R.A., Wiesner R.H., Persing D.H., Cockerill F.R., Thompson R.L. (2001). Natural history of vancomycin-resistant enterococcal colonization in liver and kidney transplant recipients. Liver Transpl..

[40] Klare I., Bender J.K., Werner G., Koppe U., Sin M.A., Eckmanns T. (2017). Eigenschaften, Häufigkeit und Verbreitung von Vancomycinresistenten Enterokokken (VRE) in Deutschland. Epi. Bull..

[41] Monteserin N., Larson E. (2016). Temporal trends and risk factors for healthcare-associated vancomycin-resistant enterococci in adults. J. Hosp. Infect..

[42] Faron M.L., Ledeboer N.A., Buchan B.W. (2016). Resistance Mechanisms, Epidemiology, and Approaches to Screening for Vancomycin-Resistant *Enterococcus* in the Health Care Setting. J. Clin. Microbiol..

[43] Van Harten R.M., Willems R.J.L., Martin N.I., Hendrickx A.P.A. (2017). Multidrug-Resistant Enterococcal Infections: New Compounds, Novel Antimicrobial Therapies?. Trends Microbiol..

[44] Yarlagadda V., Sarkar P., Samaddar S., Haldar J. (2016). A Vancomycin Derivative with a Pyrophosphate-Binding Group: A Strategy to Combat Vancomycin-Resistant Bacteria. Angew. Chem. Int. Ed. Engl..

[45] Martin E.M., Bryant B., Grogan T.R., Rubin Z.A., Russell D.L., Elashoff D., Uslan D.Z. (2018). Noninfectious Hospital Adverse Events Decline After Elimination of Contact Precautions for MRSA and VRE. Infect. Control Hosp. Epidemiol..

[46] Zachariah P., Freedberg D.E. (2019). Vancomycin use in surrounding patients during critical illness and risk for persistent colonization with vancomycin-resistant *Enterococcus*. J. Hosp. Infect..

